# Cementoblastoma Arising in the Maxilla of an 8-Year-Old Boy: A Case Report

**DOI:** 10.1155/2011/384578

**Published:** 2011-05-17

**Authors:** Hiroyuki Harada, Ken Omura, Seiki Mogi, Norihiko Okada

**Affiliations:** ^1^Oral Surgery, Department of Oral Restitution, Division of Oral Health Sciences, Graduate School, Tokyo Medical and Dental University, Tokyo 113-8549, Japan; ^2^Diagnostic Oral Pathology, Department of Oral Restitution, Division of Oral Health Sciences, Graduate School, Tokyo Medical and Dental University, Tokyo 113-8549, Japan

## Abstract

Cementoblastoma is an uncommon disease, representing only 1–8% of all odontogenic tumours. Furthermore, this tumour is especially uncommon in children, as only five cases have been reported in this age group. Here, we describe a case of cementoblastoma arising in the maxilla of an 8-year-old boy, that was treated with a partial maxillectomy. The patient's facial appearance has remained satisfactory, and the tumour has not recurred in the 9 years after the operation.

## 1. Introduction

Cementoblastoma, characterized by the formation of hard cemental tissue contiguous with a dental root, is an uncommon mesenchymal neoplasm, representing 1% to 8% of all odontogenic tumours [1–3]. Reported cases include patients from 6 to 75 years old, with the highest incidence in the second decade. This tumour is especially uncommon in patients under 10 years old, and its occurrence in the maxilla is also rare [4]. To our knowledge, only five cases of cementoblastoma in patients under the age of 10 have been reported [5–9] (Table 1). In this paper, we describe a case of cementoblastoma arising in the maxilla of an 8-year-old boy who was followed up for the extended period of 9 years. 

## 2. Case Report

**Figure 1:**
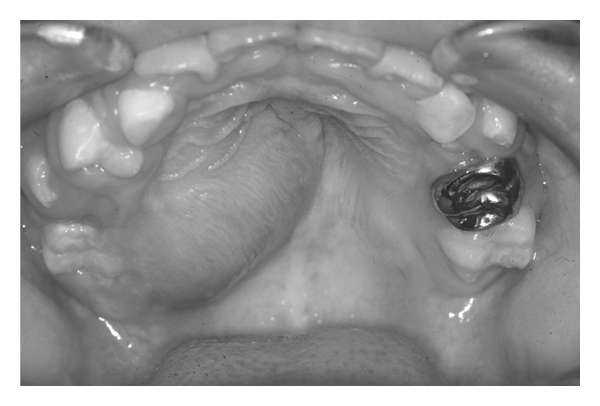
The tumour in the right hard palate.

**Figure 2:**
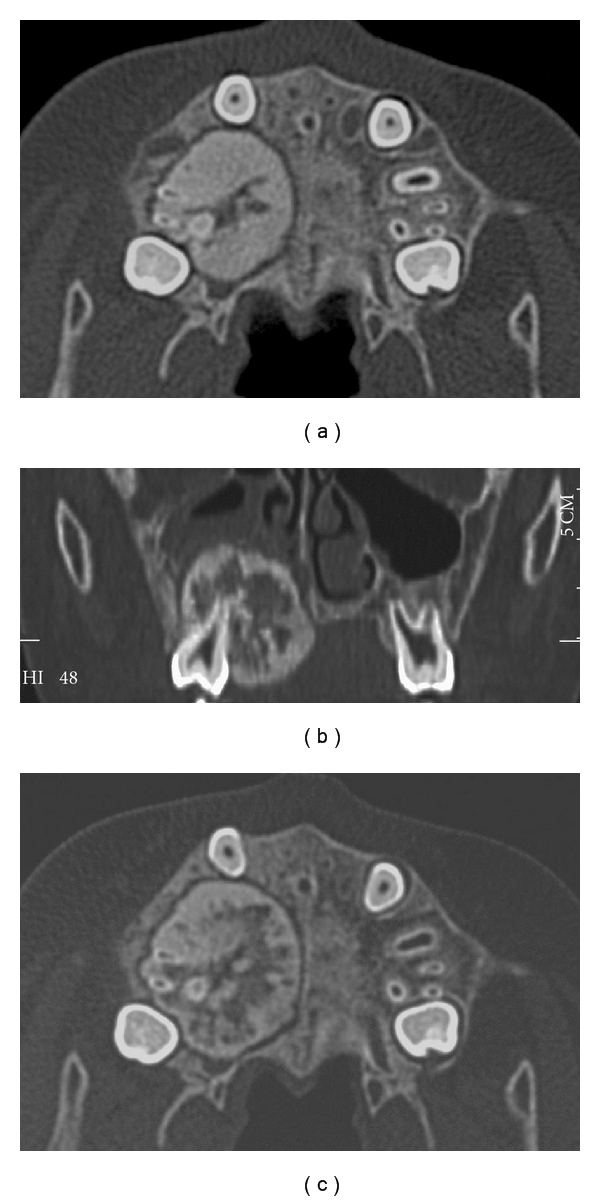
(a) The tumour contained low-density and cemental regions. (b) CT showing penetration into the nasal cavity and maxillary sinus. (c) The tumour showed rapid expansion over 2 months.

The patient was an 8-year-old boy. He was referred to our department on August 24, 2001 for evaluation of a hard mass in the right maxilla. He had noticed the asymptomatic mass 3 months before. The patient had no significant past medical history. Oral examination revealed a firm, painless mass located on the posterior hard palate, contiguous with the maxillary first molar (Figure 1). The lesion measured 3.0 cm × 2.5 cm and extended over the midline of the hard palate. The overlying mucosa showed slight redness, and the first and second premolars were displaced toward the buccal side. However, extensive mobility of the premolar and molar teeth or effect on tooth vitality determined by the electric pulp test was not observed. The patient had no nasal obstruction or difficulty with speech or chewing. A computed tomography (CT) scan showed a 2.9 cm × 2.7 cm × 2.6 cm well-circumscribed mass involving the roots of the right maxillary first molar (Figure 2(a)). The tumour consisted of low-density cemental regions that penetrated into the nasal cavity and maxillary sinus (Figure 2(b)). Bone scintigraphy showed an intense uptake in the right maxilla. The lesion was clinically diagnosed as a cementoblastoma or osteoblastoma. However, approval for immediate treatment could not be obtained by the patient and his parents. Two months after the first visit to our hospital, the oral examination and CT showed rapid expansion (0.5 cm) of the tumour (Figure 2(c)). On December 17, 2001, the patient underwent a right partial maxillectomy. 

**Figure 3:**
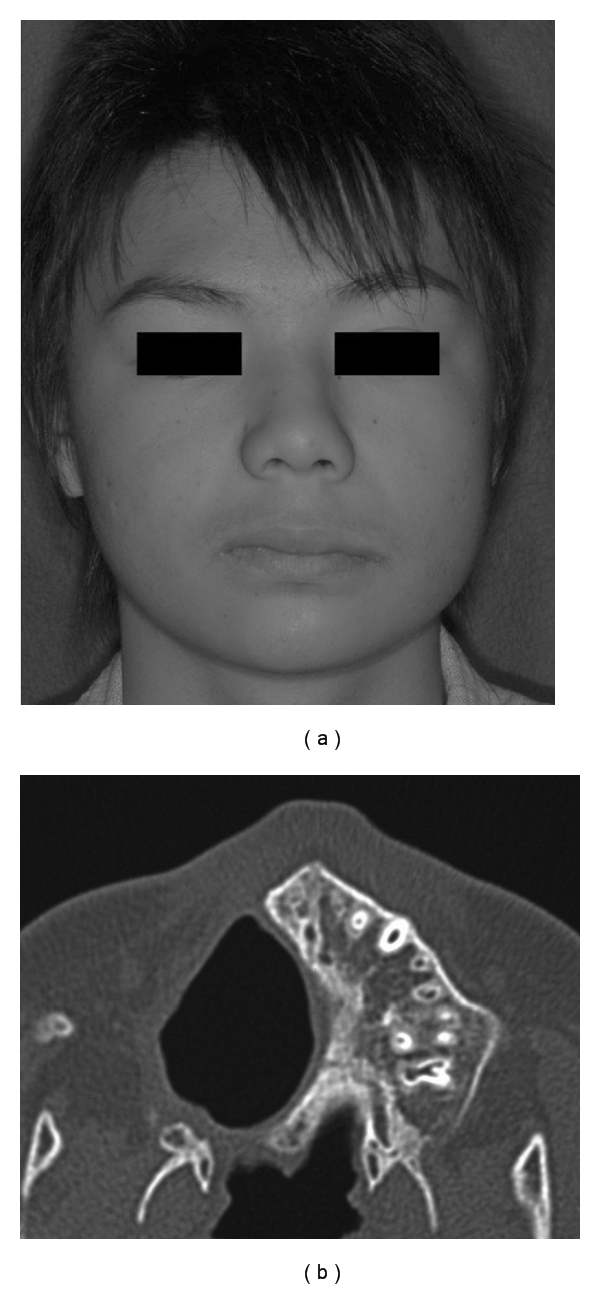
(a) Nine years after surgery, the patient has a normal appearance although the right corner of the mouth is slightly higher than the left. (b) CT taken at 9-year followup, showing the defect in the maxilla and no recurrence of tumour.

 For the operation, an incision was performed through the mucosa of the gingivobuccal sulcus and the paramedian surface of the hard palate. The periosteum of the lateral maxilla was preserved. The approach for the bone cut was through the mucosal incision. The mucosa of the residual maxillary antrum that showed inflammatory changes was curetted out, and the tetracycline ointment gauze was introduced. A previously fabricated dental obturator was set. The postoperative course was uneventful, and the dental prosthesis was frequently adjusted by our maxillofacial prosthodontist. Nine years after the operation, the right corner of the patient's mouth was slightly higher than the left corner, because of scarring, but there have been no developmental disorders of the right maxillary region (Figure 3(a)), and the tumour has not recurred (Figure 3(b)).

**Figure 4:**
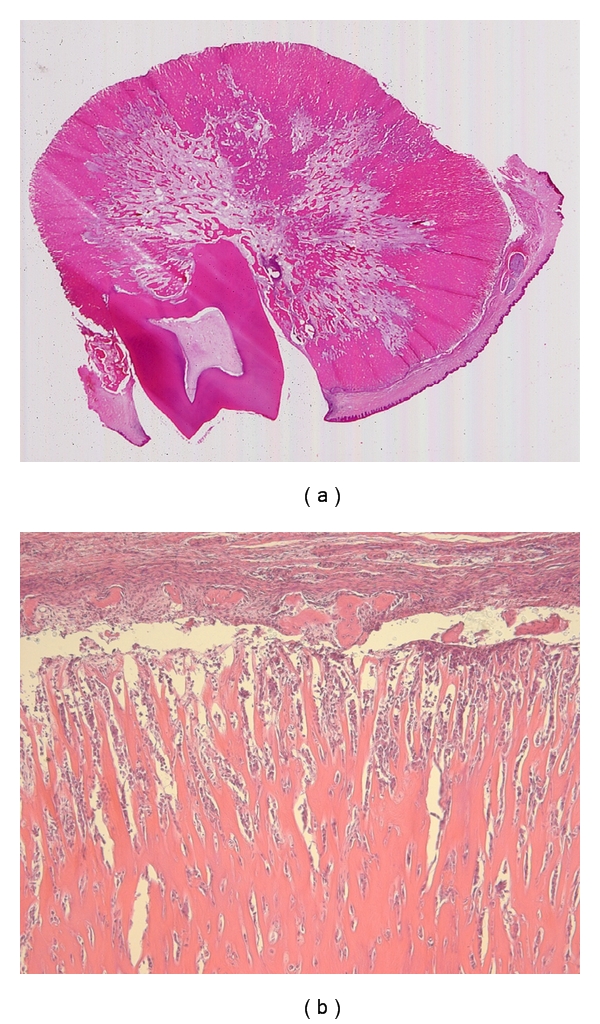
(a) The tooth root was embedded in the tumour mass. (b) The periphery of the cementoblastoma showed numerous cementoblasts forming cementum-like mineralized tissue with basophilic reversal lines (H.E.).

Macroscopically, the surgical specimen consisted of 3.5 × 3.3 × 3.0 cm, a rounded hard tissue mass confluent with the first molar tooth roots (Figure 4(a)). Microscopic examination revealed the tumour to be characterized by sheets of cementum-like tissue that contained a large number of basophilic reversal lines, surrounded by well-vascularised cellular connective tissue with abundant large cementoblasts (Figure 4(b)). The tumour was diagnosed as a cementoblastoma. 

## 3. Discussion

 Cementoblastoma has a propensity to develop in the mandible, most commonly in the molar region (80~95%). The tumour size ranges from 0.5 to 5.5 cm, and the average is 2.1 cm [1]. Cementoblastoma presents as a slowly growing, unilateral swelling of the affected bone [10]. Some patients may complain of associated pain and, occasionally, paresthesia [4]. The present case is unusual in its absence of these symptoms, large size, and its rapid increase in size, about 0.5 cm in two months. The cause of the tumour remains uncertain.

 Brannon et al. [1] reported recurrence in 15 of 69 cases of cementoblastoma (21.7%), and Sekiwa et al. [11] reported recurrence in 8 of 86 cases (9.1%). Expansion of the jaw cortex was noted in a higher percentage of recurrent tumours as compared to nonrecurrent ones [1]. Therefore, the appropriate treatment is to remove the lesion along with the affected tooth or teeth, followed by thorough curettage or peripheral ostectomy. 

Table 1 describes five cases of cementoblastoma in patients under the age of 10. In these cases, the age ranged from 6 to 8 years. In four cases, the tumour occurred in the mandible and in only one case was it in the maxilla. The tumour size ranged from 1.4 cm to 6 cm, and in four cases it was associated with a primary tooth. The treatment of all the patients in these cases was surgical enucleation, and in one case the tumour recurred (20%). 

 The treatment in most cases of odontogenic tumours in children should be the same as for those in adults. However, lesions developing in childhood grow relatively more rapidly and have unlimited growth potential [5, 6, 8, 11]; some cases require complete surgical excision and extraction of the associated tooth. Because of its rarity among patients younger than 10, there is little mention in the literature of the prognosis or the development of the jaw after the treatment of the cementoblastoma. In the present case, the right corner of the patient's mouth is raised slightly because of scarring, but the maxillary region is almost symmetrical at nine years after the operation. This good outcome may have been achieved because the periosteum in front of the maxilla was preserved, and the obturator was frequently adjusted during observation of the maxillary growth. 

 In this paper, we describe a case of cementoblastoma in an 8-year-old boy. This patient has retained a satisfactory appearance without tumour recurrence for the 9 years after his operation. 

##  Conflict of Interests

The authors declare that there are no conflict of interests. 

## Figures and Tables

**Table 1:** Clinical data for five cementoblastoma patients under age 10.

Author	Age/gender	Location of lesion	Size	Symptom (pain +/−)	Recurrence
Esguep et al. [5]	8/F	Right maxilla first molar	6.0 cm	+	+ (1 year)
Herzog [6]	7/F	Left mandible deciduous molar	1.4 cm	−	−
Papageorge et al. [7]	6/M	Central mandible deciduous incisor	4.5 cm	−	−
Zachariades et al. [8]	7/F	Right mandible deciduous molar	3.0 cm	+	−
Vieira et al. [9]	7/unknown	Left mandible deciduous molar	2.5 cm	−	−
